# Primitive Sca-1 Positive Bone Marrow HSC in Mouse Model of Aplastic Anemia: A Comparative Study through Flowcytometric Analysis and Scanning Electron Microscopy

**DOI:** 10.4061/2010/614395

**Published:** 2009-10-28

**Authors:** Sumanta Chatterjee, Pratima Basak, Prosun Das, Madhurima Das, Jacintha Archana Pereira, Ranjan Kumar Dutta, Malay Chaklader, Samaresh Chaudhuri, Sujata Law

**Affiliations:** Stem Cell Research and Application Unit, Department of Biochemistry and Medical Biotechnology, Calcutta School of Tropical Medicine, 108, C.R. Avenue, Kolkata-700073, India

## Abstract

Self-renewing Hematopoietic Stem Cells (HSCs) are responsible for reconstitution of all blood cell lineages. Sca-1 is the “stem cell antigen” marker used to identify the primitive murine HSC population, the expression of which decreases upon differentiation to other mature cell types. Sca-1^+^ HSCs maintain the bone marrow stem cell pool throughout the life. Aplastic anemia is a disease considered to involve primary stem cell deficiency and is characterized by severe pancytopenia and a decline in healthy blood cell generation system. Studies conducted in our laboratory revealed that the primitive Sca-1^+^ BM-HSCs (bone marrow hematopoietic stem cell) are significantly affected in experimental Aplastic animals pretreated with chemotherapeutic drugs (Busulfan and Cyclophosphamide) and there is increased Caspase-3 activity with consecutive high Annexin-V positivity leading to premature apoptosis in the bone marrow hematopoietic stem cell population in Aplastic condition. The Sca-1^bright^, that is, “more primitive” BM-HSC population was more affected than the “less primitive” BM-HSC Sca-1^dim ^ population. The decreased cell population and the receptor expression were directly associated with an empty and deranged marrow microenvironment, which is evident from scanning electron microscopy (SEM). The above experimental evidences hint toward the manipulation of receptor expression for the benefit of cytotherapy by primitive stem cell population in Aplastic anemia cases.

## 1. Introduction

Multipotent self-renewing HSCs are responsible for replenishing all blood cell lineages throughout the life [1, 2]. Although representing only 1 in 10 000 bone marrow cells, sophisticated methods have been developed to isolate HSCs to high purity allowing meaningful studies of candidate stem cells in vitro. Clonogenic multipotent mouse HSCs are contained within the Sca-1^+^ population of hematopoietic cells [3, 4] and Sca-1 is the most common marker often used to identify adult murine HSCs [5, 6] and can be used to isolate a nearly pure primitive HSC population [7, 8]. Sca-1 expression is regulated in a complex fashion in the hematopoietic microenvironment. As HSCs differentiate into common myeloid or lymphoid progenitors, the Sca-1 expression is downregulated [9–11]. Maintenance of these primitive Sca-1^+^ HSCs is thought to depend on specialized microenvironmental nest within the bone marrow, which has been historically called the hematopoietic inductive microenvironment [12] or “Stem Cell niche” [13]. The stem cell niche in one way maintains the hematopoiesis by secreting a large number of hematopoietic growth factors supporting proliferation, differentiation and survival of HSCs [14, 15], and in other way it maintains the quiescent stem cell pool by tightly regulating the homeostatic balance between self-renewal and differentiation [16, 17].

 In several hematological malignancies, this hematopoietic inductive microenvironment loses its controlling potential to maintain the homeostatic balance between self-renewal and differentiation of HSCs leading to either hypoplastic or hyperplastic marrow failure. Chronic hypoplastic bone marrow failure or Aplastic anemia is one of such disease characterized by an empty bone marrow, neutropenia , and a drastic decline in marrow's ability to produce healthy mature blood cells [18, 19]. The disease was first described by Ehrlich at the end of nineteenth century [20] and has been considered to involve primitive stem cell deficiency or more currently considered disorganized stem cell niche [21–24]. In most cases of Aplastic anemia, bone marrow failure is believed to result from immunologically mediated destruction of primitive hematopoietic stem and progenitor cells [25, 26]. There is also indirect evidence suggesting high rate of premature apoptosis in bone marrow hematopoietic compartment leading to a degenerative marrow in aplasia [27–30]. Although much reports are present regarding the stem cell dysregulation in Aplastic anemia, the particular evidence of the involvement of primitive HSC and HSC niche is still obscure. 

 The present study aims toward evaluating the fate/apoptosis of primitive Sca-1^+^ HSC population and bone marrow microenvironmental structure through cell surface phenotypic study by flowcytometric (FCM) analysis/caspase-3 assay, light microscopy, and scanning electron microscopic (SEM) evaluation, respectively, in experimentally induced Aplastic anemic mice following pretreatment with cytotoxic drugs like busulfan and cyclophosphamide [31, 32].

## 2. Materials and Methods

### 2.1. Animal

Inbred Swiss albino mice, thirty in number (15 animals each for normal control and experimental group) were housed in filter top cages and watered ad libitum. Twenty weeks old mice weighed to approximately 18–20 gm, received 20 mg/kg busulfan and 80 mg/kg cyclophosphamide intraperitoneally on day 0th and 28th. Controls received comparable volumes of saline [32].

### 2.2. Peripheral Blood

Twelve weeks after the second injection of Busulfan and Cyclophosphamide, approximately 300 *μ*L of blood was collected by tail vein puncture from each animal using heparinised vials. Neutrophil count, total and differential WBC count, reticulocyte, platelet count, and hemoglobin percentage were taken for the detection of whole hemogram profile.

### 2.3. Isolation of BM-HSC

Eighty-four days after the second injection for development of Aplastic anemia, animals were sacrificed to isolate the long bones (femur, tibia, fibula). Bone marrow was taken out from the above mentioned bones by flushing with RPMI 1640 media by syringe and the cells were mixed well with repeated pipetting. Stem cells were separated from the whole bone marrow population through percoll density gradient at the interface of 1.050 density [33, 34]. Cells thus prepared were washed in PBS and finally transferred to non-FBS RPMI 1640 media and the cell distribution was noted by counting the cells for the experimental group of animal.

### 2.4. Flow Cytometry (FCM) Analysis

(a) For the analysis of membrane bound Sca-1, BM-HSCs (1 × 10^6^) were incubated for 30 minutes at 37°C in dark with 2 *μ*L of phycoerythrin (PE) labeled anti-mouse Sca-1 mAb (BD-Bioscience, USA) and then washed twice in phosphate-buffered saline to wash off the excess fluorescence. Samples were analyzed by BD-FACS Callibur (Becton Dickenson, USA) using CellQuestpro software.

(b) For the analysis of apoptosis, BM-HSCs (1 × 10^6^) were incubated for 30 minutes at 37°C in dark with 2 *μ*L of PE- labeled anti-mouse Sca-1 mAb (BD-Bioscience, USA) and 5 *μ*L (diluted with binding buffer) of Fluorescein isothiocyanate- (FITC-) labeled anti-mouse Annexin V mAB (e-bioscience, Sandiego, USA). Excess fluorescence was then washed off with PBS. Samples were analyzed by BD-FACS Callibur (Becton Dickenson, USA) using CellQuestpro software.

### 2.5. Assay for Caspase-3 Activity

Intracellular caspase-3 activity of the BM-HSC were assessed by caspase-3 colorimetric assay kit (Clonetech, USA). Approximately 2 × 10^6^ BM-HSCs were lysed by incubating for 10 minutes with 50 *μ*L of chilled cell lysis buffer on ice. The cell lysate was centrifuged, the pellet discarded, and the supernatant mixed with 50 *μ*L of 2x reaction buffer/DTT mix, 5 *μ*L of 1 mM Caspase-3 substrate (DEVD-pNA; 50 *μ*M final concentration) and incubated at 37°C for 1 hour in water bath. The negative control reaction did not contain the conjugated substrate. Finally, the samples were read at 405 nm in a microplate reader.

### 2.6. Bone Marrow Smear Study

Bone marrows of the normal and experimental Aplastic groups of animals were stained by Leishman staining to observe the particular morphological changes of the bone marrow architecture in the disease concerned under light microscopy (400x magnification).

### 2.7. Sample Preparation and Scanning Electron Microscopy (SEM)

A small portion of the intact marrow tissue containing the total niche was macerated slowly in physiological manner without damaging the inner cell mass and kept in 2.8% glutaraldehyde overnight for fixation. To dry the tissue, it was repeatedly passed through 30%, 50%, 70%, 100% gradients of alcohol and finally critical point drying was done. Then after coating with gold (Au) in IB-2 ion, the coater samples went through Scanning Electron Microscopic examination using S-5330 Hitachi SEM and the results were recorded.

### 2.8. Statistical Analysis

Statistical analysis of results was performed using Students' *t*-test of the standard deviation from the mean of different data. All results were evaluated statistically by applying the SPSS-PC package (Version 9.0, SPSS, Chicago, USA). A probability of *P* < .01 was considered statistically significant.

## 3. Results

### 3.1. Peripheral Blood Hemogram

In order to ascertain the clinical status of the disease concerned, hemogram of the peripheral blood including hemoglobin, reticulocytes, WBC, polymorpho nuclear neutrophils and platelets were determined following standard laboratory techniques. The results (Table 1) showed a depressed hemoglobin level with uniformly reduced corpuscular counts, of which reticulocyte counts are significantly low (≤0.2) in Aplastic groups (0.18%) compared with the normal range (0.73%–1%). The total WBC and neutrophil counts were found to be significantly low in the diseased group (WBC 2.7 × 10^3^/*μ*L, Neutrophil 7.35%, respectively) compared to the normal counterparts (WBC 6.2 × 10^3^/*μ*L, Neutrophil 22.75%).

### 3.2. Flowcytometric Analysis

**Figure 1:**
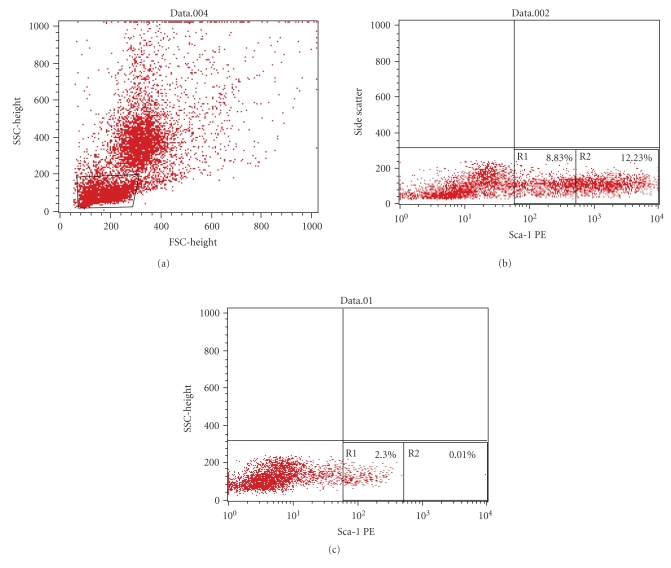
(a) Gated region of flowcytometric analysis. (b) Phenotypic characterization of Sca-1 expression by normal bone marrow cells. R-1 region indicates the Sca-1^dim^ population (8.83%). R-2 region indicates the Sca-1^bright^ population (12.23%). (c) Phenotypic characterization of Sca-1 expression by Aplastic anemic bone marrow cells. R-2 region, that is, Sca-1^bright^ population is more severely affected (0.01%) compared to the Sca-1^dim^ population in the R-1 region.

**Figure 2:**
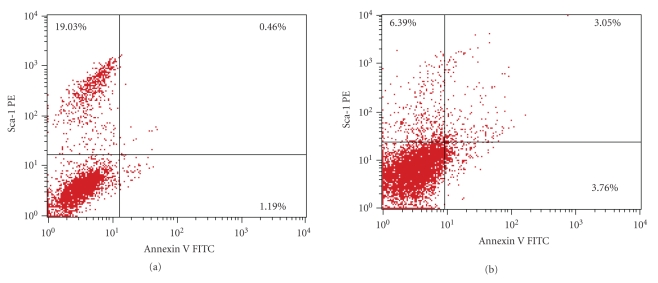
(a) Annexin-V positivity shown by Sca-1^+^ bone marrow cells in normal condition (0.46%). (b) Annexin-V positivity shown by Sca-1^+^ bone marrow cells in Aplastic anemia (3.05%) is much higher compared to the normal.

(a) Phenotypic characterization of Sca1 receptor expression by BM-HSCs indicated a highly downregulated Sca1^+^ population in Aplastic anemia induced mice. Sca1^bright^ population (R2 region) is further severely affected in Aplastic anemia (Figure 1(c)) with only 0.01% of the gated cells present in R2 region compared to 12.23% present in the normal (Figure 1(b)). Further, the R1 region denotes the Sca1^dim^ population and shows 2.30% Sca1^dim^ cells were present in Aplastic anemia compared to the 8.83% in normal.

(b) Annexin-V positivity exhibited by Sca-1^+^ BM-HSCs in Aplastic anemia (3.05%) (Figure 2(b)) was found to be significantly higher compared to the normal (0.46%) (Figure 2(a)). High Annexin-V positivity in the Sca-1^+^ BM-HSCs indicates higher rate of premature senescence in the primitive bone marrow population in Aplastic anemia.

### 3.3. Caspase-3 Activity

**Figure 3:**
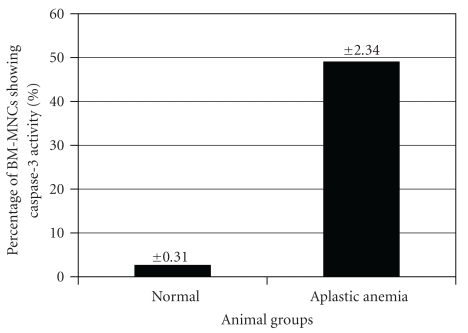
Caspase-3 activity showed by BM-HSCs in Aplastic anemia (49% ± 2.34%) (*P* < .05) was significantly higher compared to that of the normal counterpart (2.6% ± 0.31%) (*P* < .01).

Caspase-3 activity showed by BM-HSCs in Aplastic anemia (49%) was significantly higher compared to that of the normal control (2.6%) (Figure 3), further confirming the fact that the bone marrow HSCs undergo premature apoptosis in Aplastic anemia, ultimately resulting in marked decrease in the primitive Sca1^+^ population which have already been confirmed by Flowcytometric analysis.

### 3.4. Bone Marrow Architecture Study

**Figure 4:**
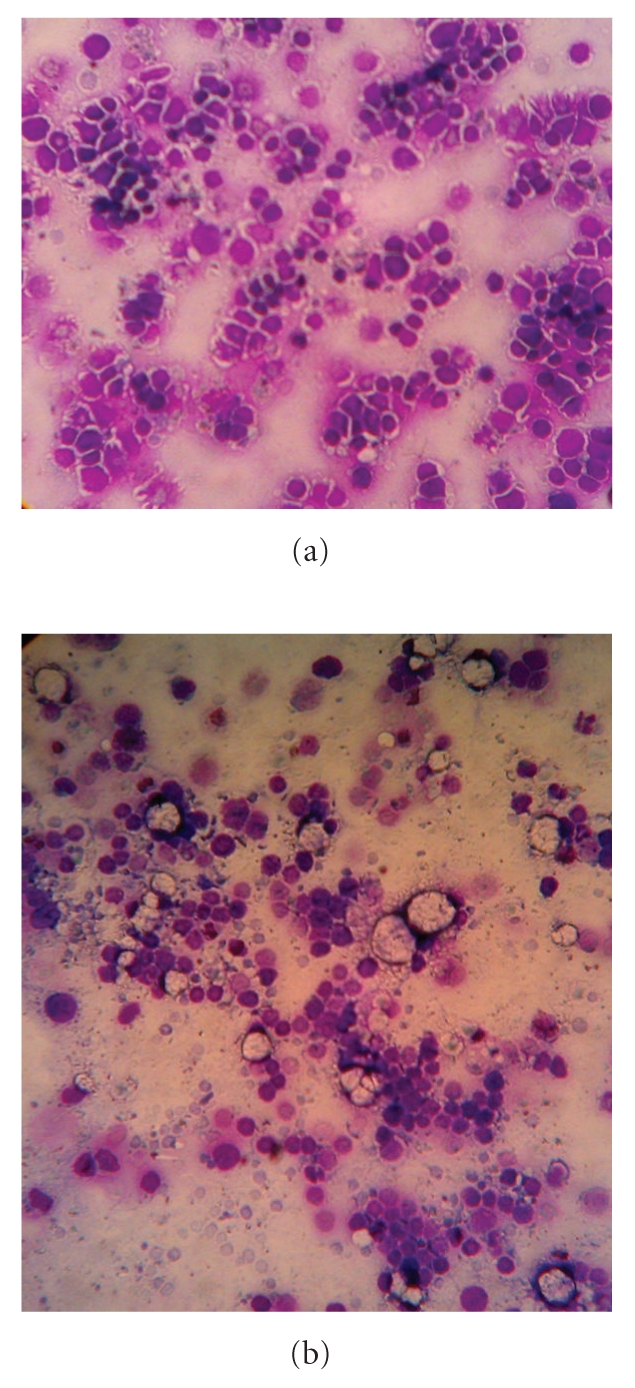
(a) Leishman staining of bone marrow smear showing densely packed cellular distribution in normal condition. (b) Leishman staining of Aplastic bone marrow smear exhibiting frequent appearance of large fat cells (adipocytes) with empty spaces.

The typical bone marrow architecture was observed in normal as well as in experimental groups by bone marrow smear study. In Aplastic anemia (Figure 4(b)), the marrow is filled with large fat cells (adipocytes) leaving scattered empty spaces in comparison with the normal (Figure 4(a)), which showed densely packed distribution of cells.

### 3.5. Scanning Electron Microscopy for Bone Marrow Microenvironmental Structure

**Figure 5:**
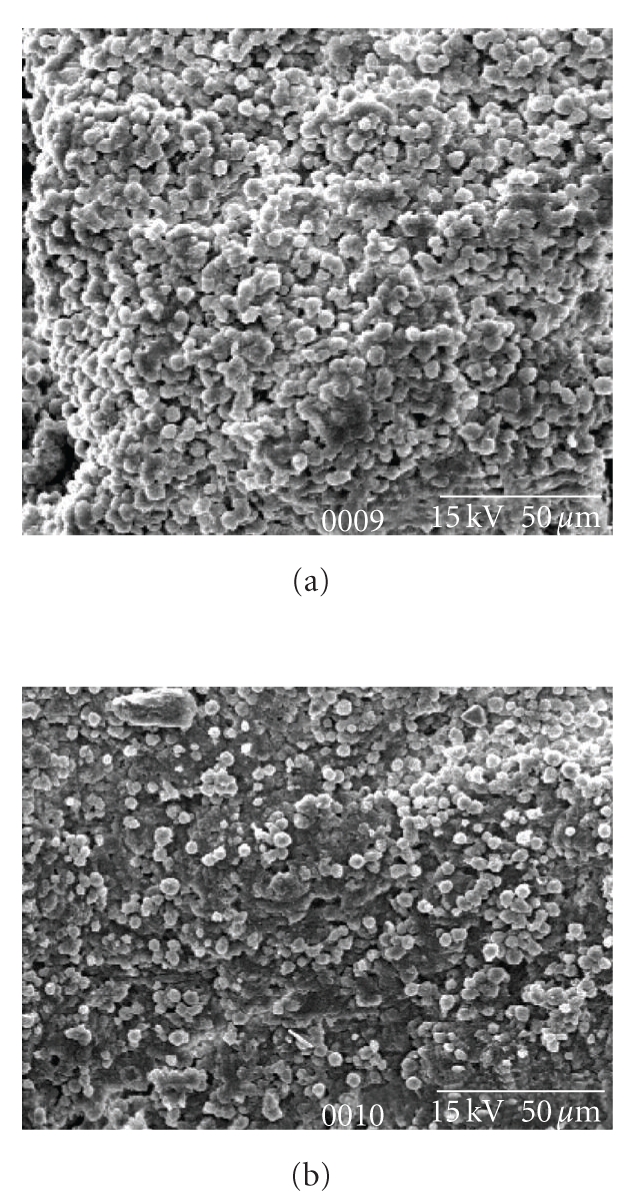
(a) Scanning electron microscopy of normal bone marrow. (b) Scanning electron microscopy of Aplastic bone marrow exhibit an overall scanty and degenerative marrow and other cellular components compared to the normal marrow.

Scanning Electron Microscopy of normal and Aplastic anemic bone marrow exhibited an overall Scanty and degenerative marrow cellularity in Aplastic anemia (Figure 5(b)) with deranged microenvironmental status and other cellular components compared to the normal marrow (Figure 5(a)).

## 4. Discussion

Bone marrow failure by benzene and related aryl hydrocarbons and alkylating agents generate catabolites that are directly toxic to stem cells and causes a drastic decline in the marrow's functional ability to produce mature blood cells [35, 36]. In our experimental setup, we used busulfan and cyclophosphamide in combination to induce bone marrow damage rather failure which is reflected in the peripheral blood hemogram. The peripheral blood pancytopenia includes neutropenia, thrombocytopenia, declined hemoglobin level, along with lesser number of reticulocytes signifies the busulfan and cyclophosphamide induced chronic hypoplastic marrow failure. At around 9 weeks after the injection of chemotherapeutic drugs, the peripheral blood started showing the hypoplastic marrow failure reflections, which was significantly evident at about 12 weeks (time of our experiment). 

 The scanty peripheral blood profile is the evidence of bone marrow failure characterized by damaged stem cell population that has been termed as “residual injury” [37, 38]. The characteristics of “residual injury” are that it is permanent, there is impairment of stem cell proliferation leading to moderate marrow failure and complete failure of stem cell proliferation may ensue leading to severe marrow failure and death [39, 40].

 To denote the “residual injury” of BM-HSCs, we investigated the Sca-1 receptor expression pattern. The bright and dim Sca-1^+^ primitive BM-HSCs depicted a typical pattern of receptor expression in Aplastic anemic condition. The Sca-1^bright^ population is more affected (0.01%) in Aplastic animals in comparison with the normal group (12.23%) rather than Sca-1^dim^ population, which showed 2.30% positivity in aplasia compared to 8.83% in normal.

 The most straight-forward explanation for these differential receptor expressions is based on the bone marrow primitive cell population damage induced by busulfan and cyclophosphamide. The Sca-1^bright^ stem cell population is the “more primitive” and the Sca-1^dim^ denotes the “less primitive” population. We hypothesize that, due to the difference in their maturity level the Sca-1^bright^, that is, “more primitive” population is getting more affected regarding their receptor expression than the Sca-1^dim^, that is, “less primitive” cell population which can resist themselves a little more due to their maturity level though both of these populations are in quiescent state, from the chemotherapeutic damage. So, the Sca-1^dim^ and Sca-1^bright^ receptor expression as well as their maturity level has significant correlation with the combined chemotherapeutic drug induced bone marrow cellular damage.

 The reduction of Sca-1 population in Aplastic anemia shows damage of primitive stem cells and their premature senescence which is further demonstrated by flowcytometric analysis using an apoptotic marker Annexin-V. Regarding the Sca-1^+^ Annexin-V^+^ population, that is, the dual positive cells, the pattern of apoptosis in the Sca-1^+^ BM-HSC is found to be significantly higher in Aplastic group (3.05%) in comparison with the normal (0.46%). So we can say that the Aplastic BM-HSC bares a much higher shift to the dual positive zone, signifying a higher apoptotic pattern of Sca-1^+^ cells.

 From the functional viewpoint, we examined the caspase-3 level in Aplastic anemic condition in comparison with the normal. The results showed inspiring interrelationship regarding the higher cellular (BM-HSCs) apoptosis in Aplastic anemic condition at the backdrop of elevated caspase-3 level which is much lower in case of normal. So after induction of aplasia by combined chemotherapeutics BM-HSCs delineated reduction of Sca-1^bright/dim^ population as well as highly elevated caspase-3 level.

Bone marrow smear study revealed a typical Aplastic anemic bone marrow feature with frequent presence of large fat cells (adipocytes) with empty spaces compared to the normal healthy marrow.

 The scanning electron microscopic picture showed lack of cell in the bone marrow of Aplastic animals that bears hollow cavity like appearance as well, which is not found in the normal bone marrow. The hollow cavities reflected the premature senescence pattern by combined chemotherapeutic induction and associated higher level of caspase-3 activity.

 So we can conclude that busulfan and cyclophosphamide induce massive destruction of BM HSCs leading to marrow hypoplasia, peripheral pancytopenia, and bone marrow microenvironmental damage [41–43] by forming deranged marrow structure with “pin-hole” views.

 Finally, the gross “residual damage” evidenced by SEM and increased caspase-3 level also severely affected the Sca-1^bright^, that is, “more primitive” BM-HSC population more than the “less primitive” BM-HSC Sca-1^dim^ depending on their maturity status.

## Figures and Tables

**Table 1:** Peripheral blood hemogram showed a depressed hemoglobin level with uniformly reduced corpuscular counts, of which reticulocyte counts are significantly low (≤0.2) in Aplastic groups compared with the normal range. The total WBC and Neutrophil counts were also found to be significantly low in the diseased group compared to the normal.

Parameters	Normal control groups (Mean ± SD)	Aplastic anemic groups (Mean ± SD)
Hemoglobin (g/dL)	15.99 ± 0.26	9.08 ± 0.47
WBC (x 10^3^/*μ*L)	6.2 ± 1.28	2.7 ± 0.68
RBC (x 10^6^/*μ*L)	8.44 ± 0.32	3.8 ± 0.86
Platelets (x 10^3^/*μ*L)	432 ± 13.82	198 ± 14.36
Reticulocyte(%)	0.89 ± 0.15	0.18 ± 0.07
Neutrophil(%)	22.75 ± 2.21	7.35 ± 2.75

## Abbreviations

BM-HSC: bone marrow hematopoietic stem cell;
AA: Aplastic anemia;
FCM: flowcytometry;
SEM: scanning electron microscopy;

## References

[1] Kondo M, Wagers AJ, Manz MG (2003). Biology of hematopoietic stem cells and progenitors: implications for clinical application. *Annual Review of Immunology*.

[2] Yang L, Bryder D, Adolfsson J (2005). Identification of Lin^−^Sca-1^+^Kit^+^CD34^+^Flt3^−^ short-term hematopoietic stem cells capable of rapidly reconstituting and rescuing myeloablated transplant recipients. *Blood*.

[3] Haylock DN, Williams B, Johnston HM (2007). Hemopoietic stem cells with higher hemopoietic potential reside at the bone marrow endosteum. *Stem Cells*.

[4] Christensen JL, Weissman IL (2001). Flk-2 is a marker in hematopoietic stem cell differentiation: a simple method to isolate long-term stem cells. *Proceedings of the National Academy of Sciences of the United States of America*.

[5] Spangrude GJ, Heimfeld S, Weissman IL (1988). Purification and characterization of mouse hematopoietic stem cells. *Science*.

[6] Okada S, Nakauchi H, Nagayoshi K, Nishikawa S-I, Miura Y, Suda T (1992). In vivo and in vitro stem cell function of C-kit- and Sca-1-positive murine hematopoietic cells. *Blood*.

[7] Matsuzaki Y, Kinjo K, Mulligan RC (2004). Unexpectedly efficient homing capacity of purified murine hematopoietic stem cells. *Immunity*.

[8] Takano H, Ema H, Sudo K, Nakauchi H (2004). Asymmetric division and lineage commitment at the level of hematopoietic stem cells: inference from differentiation in daughter cell and granddaughter cell pairs. *The Journal of Experimental Medicine*.

[9] Akashi K, Traver D, Miyamoto T (2000). A clonogenic common myeloid progenitor that gives rise to all myeloid lineages. *Nature*.

[10] Trevisan M, Iscove NN (1995). Phenotypic analysis of murine long-term hemopoietic reconstituting cells quantitated competitively in vivo and comparison with more advanced colony- forming progeny. *The Journal of Experimental Medicine*.

[11] Kondo M, Weissman IL, Akashi K (1997). Identification of clonogenic common lymphoid progenitors in mouse bone marrow. *Cell*.

[12] Curry JL, Trentin JJ, Wolf N (1967). Hemopoietic spleen colony studies. II. Erythropoiesis. *The Journal of Experimental Medicine*.

[13] Schofield R (1978). The relationship between the spleen colony-forming cell and the haemopoietic stem cell. *Blood Cells*.

[14] Kopp H-G, Avecilla ST, Hooper AT, Rafii S (2005). The bone marrow vascular niche: home of HSC differentiation and mobilization. *Physiology*.

[15] Rafii S, Mohle R, Shapiro F, Frey BM, Moore MAS (1997). Regulation of hematopoiesis by microvascular endothelium. *Leukemia and Lymphoma*.

[16] Domen J, Weissman IL (1999). Self-renewal, differentiation or death: regulation and manipulation of hematopoietic stem cell fate. *Molecular Medicine Today*.

[17] Wilson A, Trumpp A (2006). Bone-marrow haematopoietic-stem-cell niches. *Nature Reviews Immunology*.

[18] Young NS (2002). Acquired Aplastic anemia. *Annals of Internal Medicine*.

[19] Young NS, Kasper DL, Braunwald E, Fauci AS, Hauser SL, Longo DL, Jameson JL (2005). Aplastic anemia, myelodysplasia, related bone marrow failure syndromes. *Harrisons Principles of Internal Medicine*.

[20] Ehrlich P (1888). Uber einen Fall Von Anime mit Bemerkungen uber regenerative veraenderungen des Knochenmarks. *Charite-Annalen*.

[21] Maciejewski JP, Selleri C, Sato T (1996). A severe and consistent deficit in marrow and circulating primitive hematopoietic cells (long-term culture-initiating cells) in acquired Aplastic anemia. *Blood*.

[22] Scopes J, Bagnara M, Gordon-Smith EC (1994). Haemopoietic progenitor cells are reduced in Aplastic anaemia. *British Journal of Haematology*.

[23] Yoshida K, Miura I, Takahashi T (1983). Quantitative and qualitative analysis of stem cells of patients with Aplastic anaemia. *Scandinavian Journal of Haematology*.

[24] Law S, Chaudhuri S (2007). Niche theory, stem-stromal imbalance and Aplastic anaemia. *Journal of Stem Cells*.

[25] Juneja HS, Lee S, Gardner FH (1989). Human long-term bone marrow cultures in Aplastic anemia. *International Journal of Cell Cloning*.

[26] Testa N, Heardy J, Molineux G, Testa NG, Gale RP (1988). Long term bone marrow damage after cytotoxic treatment: stem cells and microenvironment. *Hematopoiesis*.

[27] Krammer PH (2000). CD95’s deadly mission in the immune system. *Nature*.

[28] Young NS (2000). Hematopoietic cell destruction by immune mechanisms in acquired Aplastic anemia. *Seminars in Hematology*.

[29] Maciejewski JP, Selleri C, Sato T, Anderson S, Young NS (1995). Increased expression of Fas antigen on bone marrow CD34^+^ cells of patients with Aplastic anaemia. *British Journal of Haematology*.

[30] Li W, Fu J, Wang F (2004). Distinct overexpression of Fas ligand on T lymphocytes in Aplastic anemia. *Cellular & Molecular Immunology*.

[31] Chen J (2005). Animal models for acquired Bone marrow failure syndromes. *Clinical Medicine & Research*.

[32] Marley A, Blake J (1974). An animal model of chronic Aplastic marrow failure. I. Late marrow failure after busulfan. *Blood*.

[33] Law S, Maiti D, Palit A (2001). Facilitation of functional compartmentalization of bone marrow cells in leukemic mice by biological response modifiers: an immunotherapeutic approach. *Immunology Letters*.

[34] Law S, Maiti D, Palit A, Chaudhuri S (2003). Role of biomodulators and involvement of protein tyrosine kinase on stem cell migration in normal and leukaemic mice. *Immunology Letters*.

[35] Hugo C, Malaspina, Richard JO, Goldman L, Ausiello D (2007). Anemia and related disorders. *Cecil Textbook of Medicine*.

[36] Trainor KJ, Morley AA (1976). Screening of cytotoxic drugs for residual bone marrow damage. *Journal of the National Cancer Institute*.

[37] Pugsley CAJ, Forbes IJ, Morley AA (1978). Immunologic abnormalities in an animal model of chronic hypoplastic marrow failure induced by busulfan. *Blood*.

[38] Twomey JJ, Douglass CC, Sharkey O (1973). The monocytopenia of Aplastic anemia. *Blood*.

[39] Morley A, Trainor K, Blake J (1975). A primary stem cell lesion in experimental chronic hypoplastic marrow failure. *Blood*.

[40] Morley A, Blake J (1974). Haemopoietic precursor cells in experimental hypoplastic marrow failure. *Australian Journal of Experimental Biology and Medical Science*.

[41] Botnick LE, Hannon EC, Hellman S (1979). A long lasting proliferative defect in the hematopoietic stem cell compartment following cytotoxic agents. *International Journal of Radiation Oncology, Biology, Physics*.

[42] Fitchen JH, Deregnaucourt J, Cline MJ (1981). An in vitro model of hematopoietic injury in chronic hypoplastic anemia. *Cell and Tissue Kinetics*.

[43] Millar JL, Hudspith BN, Blackett NM (1975). Reduced lethality in mice receiving a combined dose of cyclophosphamide and busulphan. *British Journal of Cancer*.

